# Epidemiological, clinical, diagnostic and economic features of an immigrant population of chronic schistosomiasis sufferers with long-term residence in a non-endemic country (North Metropolitan area of Barcelona, 2002-2016)

**DOI:** 10.1371/journal.pone.0185245

**Published:** 2017-09-27

**Authors:** Sílvia Roure, Lluís Valerio, Olga Pérez-Quílez, Gema Fernández-Rivas, Octavio Martínez-Cuevas, Albert Alcántara-Román, Diego Viasus, M. Luisa Pedro-Botet, Miquel Sabrià, Bonaventura Clotet

**Affiliations:** 1 North Metropolitan International Health Unit PROSICS, Hospital Universitari Germans Trias i Pujol, Universitat Autònoma de Barcelona, Badalona, Barcelona, Spain; 2 Infectious Diseases Unit, Internal Medicine Department, Hospital Universitari Germans Trias i Pujol, Universitat Autònoma de Barcelona, Badalona, Barcelona, Spain; 3 North Metropolitan International Health Unit PROSICS, Servei Atenció Primària, Santa Coloma de Gramenet, Barcelona, Spain; 4 Department of Microbiology, Hospital Universitari Germans Trias i Pujol, Universitat Autònoma de Barcelona, Badalona, Barcelona, Spain; 5 Health Sciences Division, Faculty of Medicine, Hospital Universidad del Norte and Universidad del Norte, Barranquilla, Colombia; 6 AIDS Research Institute-IrsiCaixa, Hospital Germans Trias i Pujol, Universitat Autònoma de Barcelona, Badalona, Spain; Universita degli Studi di Parma, ITALY

## Abstract

**Background:**

Schistosomiasis, one of the neglected tropical diseases (NTD) listed by the WHO, is an acute and chronic parasitic disease caused by blood flukes (trematode worms) of the genus *Schistosoma*. Complications of long-term infestation include liver cirrhosis, bladder tumors and kidney failure. The objective of this study was to carry out a clinical and epidemiological characterization of a schistosomiasis-diagnosed immigrant population with long-term residencein the EU as well as to evaluate the diagnostic methods available to date.

**Methods and results:**

A total of 61 individuals with *Schistosoma* infection who received medical attention between June 2002 and June 2016 at the North Metropolitan International Health Unit in Barcelona (Catalonia, Spain), were included in the study. All patients were sub-Saharan African immigrants. The majority were male (91.8%) with a median age of 34 years. Symptoms attributable to infection such as haematuria, abdominal pain and dysuria were recorded in up to 90% of patients. The percentage of eosinophils decreased amongst older patients (p = 0.002) and those with symptoms associated with urinary tract infections (p = 0.017). Serology was used for diagnosis in 80.3% of the cases, with microscopic examination showing the remaining 9.8% positive for parasite eggs. Direct microbiological diagnosis was more useful in patients with less than 5 years of residence in the EU (p = 0.05). Chronic complications were present in 22 (36%) of the patients, with renal failure affecting 20 (33%). Of these 20, 6(10%) developed terminal renal failure and required hemodialysis, while 3 (5%) received a renal transplantation.

**Conclusion:**

Morbidity associated with chronic long-term schistosomiasis is frequent among African immigrants in non-endemic countries. Better diagnostic tools and appropriate early treatment would prevent the development of visceral damage. Thorough screening in selected patients would also be useful to avoid chronic complications.

## Introduction

Schistosomiasis is an endemic helminthiasis caused by trematodes of the genus *Schistosoma*. The main disease-causing species are *Schistosoma mansoni (Africa and South America)*, *S*. *haematobium (Africa and the Middle East)*, and *S*. *Japonicum (East Asia)*. At the present time more than 74 countries worldwide are considered endemic for the disease and around 200 million individuals are infected, especially in sub-Saharan Africa. Beyond its public health implications, the infection is regarded as one of the tropical parasitic diseases with greatest socioeconomic impact [1].

Chronic schistosomal disease mainly affects individuals with long-standing infections. Immunopathological reactions to schistosome eggs trapped in the tissues lead to inflammatory and obstructive disease in either the urinary system (*S*. *haematobium*) or the digestive tract such as with hepatosplenic inflammation and even liver fibrosis (*S*. *mansoni*, *S*. *japonicum*) [2]. Despite the availability of simple and effective diagnostic tests and treatments, chronic disease is rarely symptomatic and can often lead to tissue damage resulting in severe end-stage renal failure, cirrhosis, pulmonary hypertension or, more rarely, neurological complications.

The continuous flow of immigrants from schistosomiasis-endemic countries to the European Union has inevitably included a certain number of chronically infected individuals. The usual first-step diagnostic test is based on the microscopic identification of *Schistosoma* eggs either in feces or urine, a very useful method when the parasite load is high [3]. In non-endemic countries, nevertheless, the positive predictive value of such direct techniques decreases noticeably owing to the low parasite load of long-term- infections and the lack of microscopy-experienced staff. To date, the absence of a true gold standard for quantitative correlations to actual worm burden remains a significant challenge [4].

As a result, diagnosis among immigrant populations is usually delayed and performed only when complications are already established, thus complicating the management and prognosis of the disease as well as the effectiveness of treatment. Thus active screening of the population considered at risk and the application of adequate diagnostic methods are the key to improving the prognosis of these long-term infected patients. Moreover, immediate and pro-active attention to cases could lead to substantial economic savings by avoiding organ end-stage diseases, transplants and other costly sequelae.

## Materials and methods

### Study population

From June 2002 to June 2016, information about all individuals diagnosed with a *Schistosoma* infection at the North Metropolitan International Health Unit (Barcelona, Catalonia, Spain) was recorded and included in the study. This center serves a population of approximately 400,000, of which about 12,900 (21%) are immigrants from high-endemic African countries. It belongs to the Institut Català de la Salut, a public health service, so medical visits are easily accessible and free of charge. Most of the patients were referred by family physicians, while others came from various hospital services. Schistosomiasis was diagnosed through a) a positive identification of parasite eggs in stool or urine samples, b) the presence of specific antibodies detected in blood or c), exceptionally, high clinical and/or radiological suspicion.

From May 2015, every patient complying with the following two major criteria and two or more minor criteria was systematically tested for schistosomiasis. The major criteria were a) long-term or frequent presence in an endemic country because of either or frequent travel, and b) prior history of freshwater river, lake or pond exposure. Minor criteria were any signs or symptoms suggestive of *Schistosoma* infection such as eosinophilia, haematuria, dysuria, recurrence urinary tract infections, renal failure, nephritic colic, abdominal pain, rectal bleeding, chronic hepatopathy or transaminitis of unknown origin, sterility, ictus/myelitis, urinary tract involvement, or liver involvement. The pathological antecedents and blood test data of the patients were collected in the shared intranet medical history (primary care, tertiary-level hospital and laboratory) of the *Institut Català de la Salut*. Recurrent urinary tract infections were defined as >2 urinary tract infections (urinalysis either by microscopy or by dipstick and/or urine culture with susceptibility data) and were recorded in the clinical history. Renal insufficiency were defined as decreased estimated glomerular filtration rate (< 60ml/min/1.73m^2^) or elevated blood creatinine (> 1.3mg/ml). Sterility was defined as inability of a couple to conceive after 12 months of regular intercourse without use of contraception.

Patients with positive serology but reporting previous treatment with praziquantel were excluded from the study.

Data was collected and analyzed in an anonymous way always respecting the usual confidentiality requirements. Written informed consent was obtained from all patients or their guardians. This study was approved by the Clinical Research Ethics Committee at our institution (“Comité Ético de Investigación Clínica”, CEIC).

### Diagnostic tests and management

Between June 2002 and April 2009, the only available diagnostic tool was a direct microscopy identification for parasite eggs in feces (three fixed stool samples in formalin) or urine collected in 24 hours). All samples were sent to the Microbiology Department where stool samples were processed by formalin-ethyl acetate sedimentation concentration while urine samples were centrifuged at 400 × *g*. Wet mount was performed to detect Schistosoma eggs, especially *S*. *haematobium* in urine and *S*. *mansoni* in stool samples.

From May 2009, serum samples were sent to the Centro Nacional de Microbiología (CNM) a center belonging to the *Instituto de Salud Carlos III* in Majadahonda, Madrid, Spain, to be tested for *Schistosoma* by an ELISA Novalisa^™^ IgG (Novatec Inmunodiagnostica GmbH, Germany) immunoassay [5].

An abdominal and urinary tract ultrasonography was performed on all those patients who showed positive for schistosomiasis. Patients with confirmed infection were treated with praziquantel 60mg/kg orally given in two divided doses for one day. A repeat course of treatment was given two weeks after the initial one. A prior treatment with steroids was given when neurological complications were present.

The patients were followed-up with new samples of feces/urine, a complete blood count, blood chemistry and abdominal ultrasonography, after six months of treatment.

### Statistical analysis

The variables assessed were age, sex, country of origin, length of residence in the EU (years), eosinophil count (with eosinophilia if > 500 cells/μL or absolute count > 8%), known antecedent of schistosomiasis (yes/no), presence of haematuria during childhood (yes/no), presence of symptoms (yes/no), type of symptoms, complications, diagnostic test (direct microscopy, serology or imaging test), abdominal ultrasonography (normal, abnormal), identified *Schistosoma* species, correct treatment with praziquantel (yes/no), favorable clinical course after treatment (yes/no), normal eosinophil count after treatment (yes/no) and family checking (yes/no).

Categorical variables and continuous data were reported as percentages and mean ± standard deviation (SD), respectively. To detect significant differences between groups, we used the chi-squared or Fisher’s exact test for categorical variables, and the Student t test or Mann–Whitney U test for continuous variables, when appropriate. Statistical significance was established at α = 0.05. All reported p values are two-tailed. Statistical Package for the Social Science for Windows (SPSS, version 20; Chicago, Illinois, USA) was used for the statistical analysis.

## Results

During the study period, 61 individuals were diagnosed with *Schistosoma spp*. infection. Their socio-demographic and clinical characterization is displayed in “Tables 1 and 2”. From the beginning of the screening period, 34 individuals complied with the screening criteria and, of them, 29 (85%) had a positive serology for *Schistosoma spp*. Urogenital schistosomiasis was the main type of affliction, with 45(73.8%) patients reporting having had macrohaematuria episodes during their childhood. Serious complications were recorded in 22 (36%) patients, 20 chronic renal failure (32.8%) being the most severe complication followed by 2 patients with ischemic cerebrovascular stroke (3.3%). A total of 6 patients with renal failure required hemodialysis, and 3 underwent kidney transplantation (two patients of these requiring two successive transplantations). The parasitic diagnosis in all cases was subsequent to transplantation.

**Table 1:** Socio-demographic and clinical characteristics of *Schistosoma spp* cases (n = 61).

Variable	N(%)
**Mean age, years (range)**	34 (31–42)
**Sex**	
**Male**	56 (91.8)
**Female**	5 (8.2)
**Country origin**	
**Senegal**	23 (37.7)
**Mali**	14 (23)
**Gambia**	11 (18)
**Nigeria**	4 (6.6)
**Other**	9 (14.7)
**Mean Length of residence, years (range)**	9 (7-11y)
**Eosinophil count**	
**Mean(range)**	580 (200–800)
**Median**	10 (4–13)
**According count**	31 (50.8)
**According percentage**	34 (55.7)
**Previous *Schistosoma spp*. infection**.	
**No**	49 (80.3)
**Yes**	3 (4.9)
**Childhood haematuria**	
**Yes**	45 (73.8)
**Symptoms**	
**Presence**	55 (90)
**Absence**	6 (10)
**Diagnostic test**	
**Microscopy**	6 (9.8)
**Serology**	49 (80.3)
**Clinical findings**	2 (3.3)
**Clinical and radiological findings**^a^	4 (6.5)
**Renal abdominal Ultrasound (n = 37)**	
**Abnormal**	23 (62.1)
**Urinary tract involvement**	19 (31.1)
**Liver involvement**	6 (12)
**Schistosoma type**	
**Urogenital**	34 (55.7)
**Abdominal**	8 (13.1)
**Unknown**	19 (31.1)
**Praziquantel treatment**	
**Yes**	46 (75)
**Improvement transaminase serum levels** after treatment **(n = 14)**	10 (71)
**Improvement eoshinophil** count after treatment **(n = 24)**	21 (87.5)
**Primary Care Phisician (n = 57)**	14 (23)

^a^ urinary tract involvement.

**Table 2:** Type of symptoms and complications.

Variable (n)	N (%)
**Dysuria (59)**	30 (49.2)
**Haematuria (60)**	44 (72.1)
**Recurrent urinary tract infections (58)**	23 (37.7)
**Renal colic (58)**	8 (13.1)
**Abdominal pain (58)**	31 (50.8)
**Diarrhea (59)**	5 (8.2)
**Sterility (26)**	5 (8.2)
**Renal insufficiency (59)**	20 (32.8)
**Median creatinine level (mg/dl)**	1.4 (1.39–2.06)
**Hemodialysis (60)**	6 (9.8)
**Renal transplant (60)**	3 (4.9)
**Ischemic cerebrovascular stroke (60)**	2 (3.3)

Serology was the most sensitive diagnostic test. A list of the diagnostic tools used is shown in “Table 3”.

**Table 3:** Diagnostic test.

Test	N (%)
**Dipstick urine test (49)**	23 (46.9)
**Eggs in urine (52)**	4 (7.7)
**Eggs in stool (41)**	2 (4.8)
**ELISA serology**	49 (80.3)
**Clinical suspicion**	2 (3.3)
**Clinical + radiological suspicion**^a^	4 (6.5)

^a^ urinary tract involvement.

Although family physicians had suspected the presence of parasitic infection and initiated the diagnostic process in 23% of cases, diagnosis was achieved in none of them. A total of 46 (75%) patients successfully completed the treatment with praziquantel, the majority of failures to complete being due to absence in follow-up visits. Hepatitis B coninfection with superficial antigen Hepatitis B (HBsAg+) was identified in 12 (19.7%).

**Fig 1:**
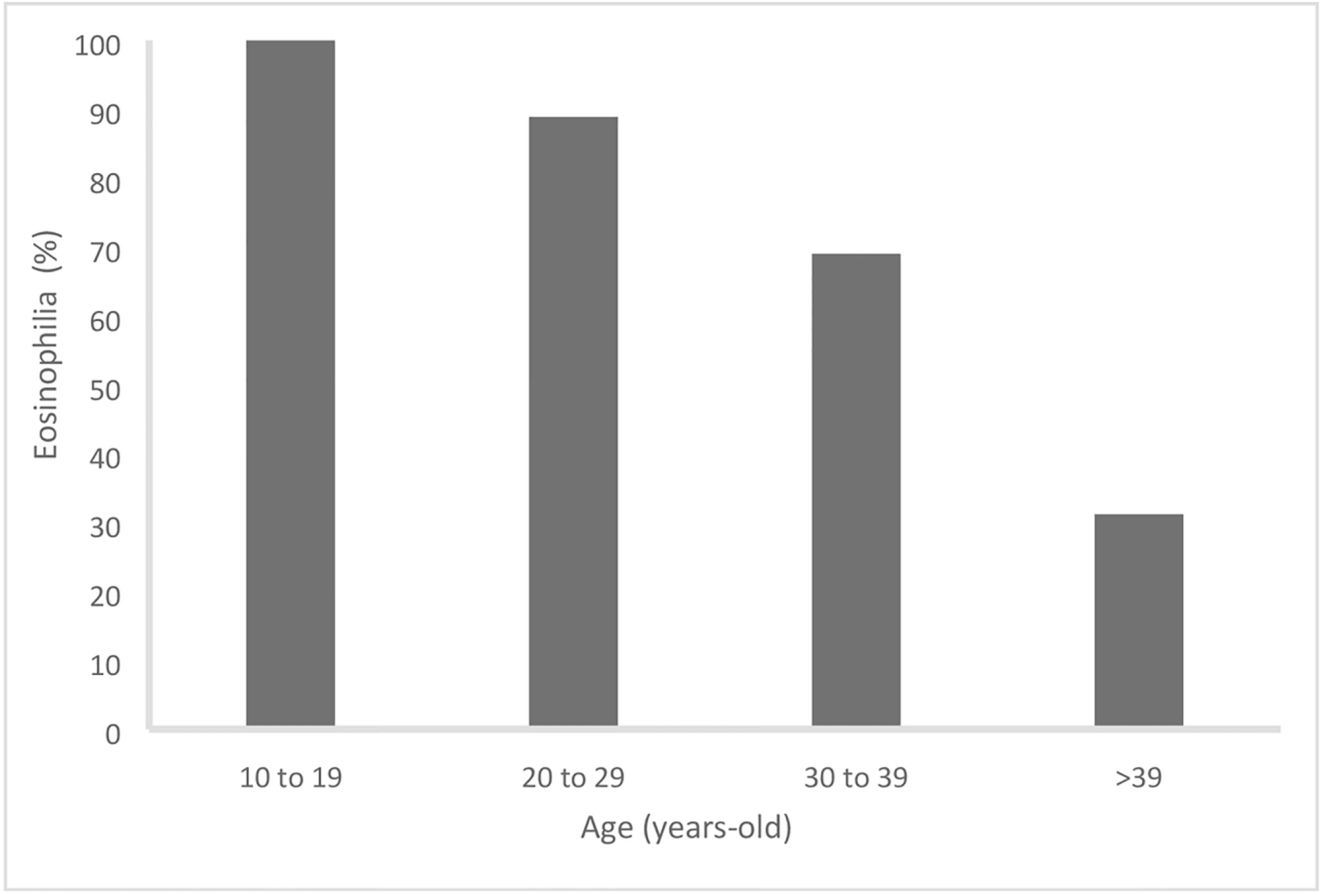
Age and eosinophil count at the time of diagnosis of *Schistosoma spp* (p = 0.002).

According to bivariate analysis, a negative relationship exists between both the eosinophil counts and eosinophil percentages and the older patients (p = 0.002 “Fig 1”, p = 0.003 and respectively). In addition, older patients showed higher creatinine levels and lower levels of eosinophilia (p = 0.029). They were also more symptomatic, had more urine infections (both p = 0,017) and tended to have more renal colic (p = 0.069). Patients with HBV coinfection had higher but not significantly elevated transaminase serum levels.

Direct microscopic examination for ova in urine or feces was a more useful method in patients who had been residing in the EU for less than 5 years, while serology testing was a clearly more effective method among patients who had resided 5 years or more in the EU (p = 0.05).

Regarding area of origin, Western Africa (especially Mali and Senegal) was the main source of infection, as shown in “Table 4”. In fact, the study population included no patients native to other endemic areas such as Brazil, Yemen or Southeast Asia. The eosinophil count was significantly higher in patients from Gambia compared with those from Senegal (90.9% vs 36.4%, p = 0.003). Abnormal ultrasound images were more frequently found in patients from Nigeria than among patients from Senegal, Mali or Gambia (p = 0.005). Elevation of transaminase serum levels was higher in patients from Nigeria but this difference was not statistically significant.

**Table 4:** Comparative analysis of variables according to patients’ country of origin.

	Gambia N = 11	Mali N = 14	Senegal N = 23	Nigeria N = 4		Other N = 9N	p^a^
**Eosinophilia**	9 (81.8%)	9 (64.2%)	11 (47.8%)	2 (50%)	6 (66.7%)		0.44
**Transaminitis**	5 (45.5%)	3 (21.4%)	7 (30.4%)	4 (100%)	3 (33.3%)		0.09
**Renal colic**	0 (0%)	3 (21.4%)	5 (21.7%)	0 (0%)	0 (0%)		0.06
**RecurrenceUTI**	1 (9%)	7 (50%)	11 (47.8%)	2 (50%)	2 (22.2%)		0.08
**Dysuria**	2 (18.1%)	7 (50%)	16 (69.5%)	2 (50%)	4 (44.4%)		0.09
**Haematuria**	6 (54.5%)	12 (85.7%)	17 (73.9%)	3 (75%)	6 (66.6%)		0.23
**Abnormal renal US**	9 (81.8%)	5 (35.7%)	11 (47.8%)	4 (100%)	9 (100%)		0.0050
***S*. *haematobium* (n = 34)**	6 (75%)	9 (100%)	14 (82.4%)	2 (50%)	3 (75%)		
***S*.*mansoni* (n = 38)**	2 (25%)	0 (0%)	3 (17.6%)	2 (50%)	3 (75%)		

**Note**:

^**a**^ Statistical significance was set at α = 0.05;

UTI: urinary tract infections; US: ultrasound.

## Discussion

The continuous flow of immigrants from endemic countries mainly in sub-Saharan Africa has conditioned the emergence of chronic parasitosis in the EU. Nevertheless, due to low symptomatology, diagnosis of these imported infections is often delayed and follow-up is often difficult owing to the frequency of changes in job and/or residence among immigrants. Frequently, diagnosis does not occur until disease has reached advanced stages with chronic liver disease or renal failure irremediably established. Other authors have already called attention to the presence of long-lasting undiagnosed schistosomiasis cases among immigrant populations established in Europe, with Senegal, the Gambia, Mali and Nigeria being the most common countries of origin [6–8]. Despite such words of warning, however, literature dealing with chronic schistosomiasis in the EU is scarce, with most studies focusing on cases involving newly acquired schistosomiasis imported by travelers rather than long-term infection [9–11]. In France, a European country with a high percentage of sub-Saharan immigrants, a systematic survey of intestinal parasite infections in an immigrant population found *Schistosoma haematobium* to be the most dangerous parasitic disease [12].

It is essential for local medical services to be able to detect signs and symptoms of schistosomiasis in such patient populations who are living far outside endemic areas. The symptoms of chronic infection often have an insidious onset and as a result, if there is no clinical suspicion, diagnosis can be late and performed when complications are already present [13].

In our study, up to 90% of the patients had symptoms attributable to the infection. Although family physicians started the diagnostic process in 23% of the cases reported, in none of these, schistosomiasis was finally diagnosed in the primary care centers. The only diagnostic test available to these primary care doctors was direct microscopy examination of urine and/or stools, which is not particularly sensitive in patients with chronic infection and low parasitic load. This technique lacks sensitivity and does not take into account day-to-day variation in egg output [14]. In other words, primary care services tend to lack proper diagnostic tools, a situation that precludes the possible implementation of any screening program. In the present study, the diagnosis was always carried out by a specialized international health unit and was based first on a high clinical suspicion and second on a proactive search for symptoms using indirect methods, mainly serology. Had the primary care services been endowed with a proper serologic test, more patients might have been identified earlier. With regard to the situation in Catalonia, the geographic area covered by the present study, recommendations along these lines had already been made by the Catalan Society of Primary Health, which called for the availability of diagnostic tools to enable the direct identification of schistosome eggs [15]. This study points to two other factors which can be used to identify risk and thus improve the predictive value of serology, namely personal antecedents of macrohaematuria (which affected 74% of the population reported here) and dipstick test results positive for blood (47%) [16,17]. Also, echographic exploration is able to show organ abnormalities in a substantial proportion of patients, and we recommend that this innocuous exploration should be included in the basic diagnostic process.

**Fig 2:**
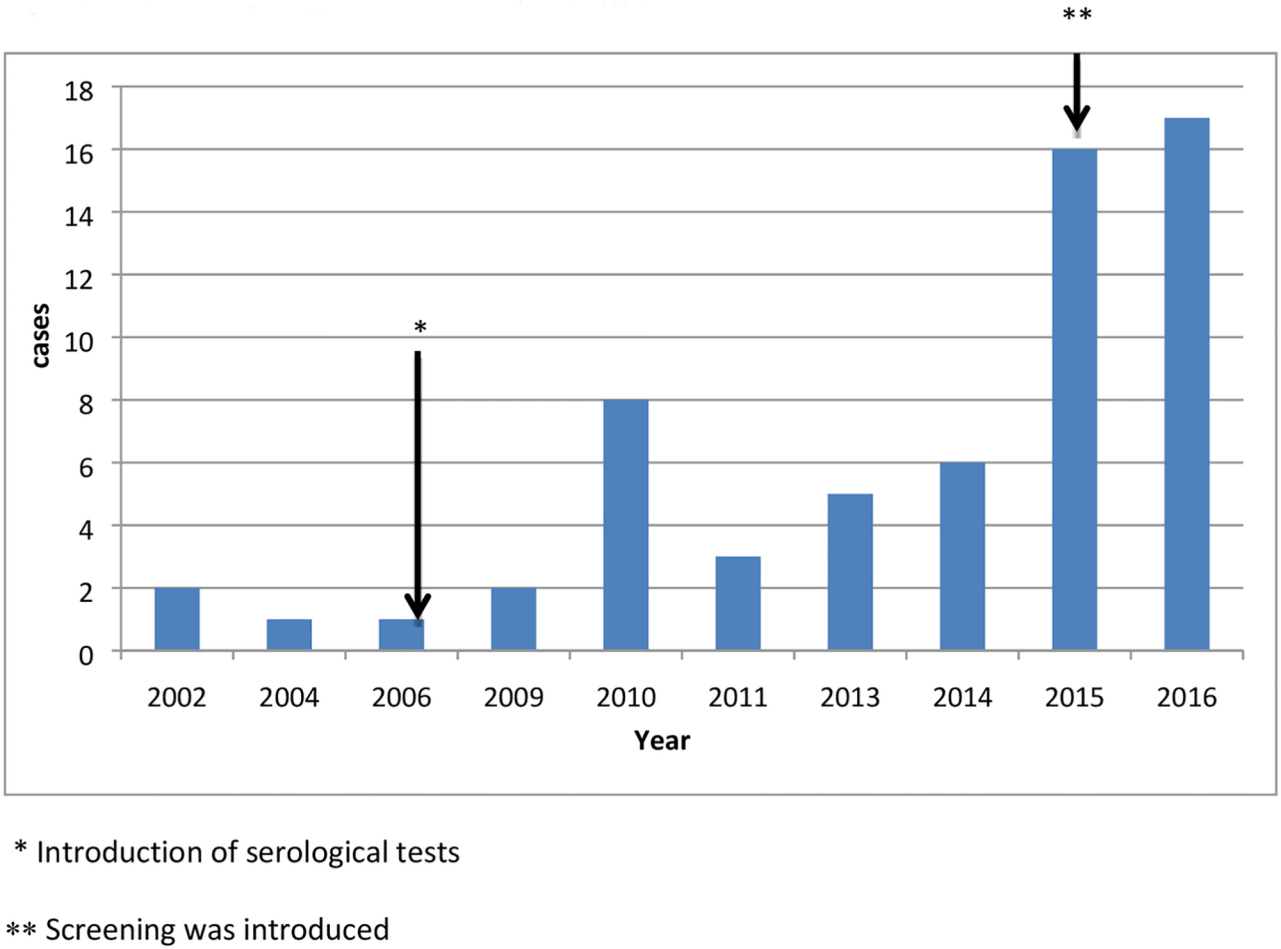
Cases of Schistosomiasis according to year of diagnosis.

Within a period of 14 years, 61 cases of schistosomiasis were identified. From 2002 to 2008 the diagnoses were based on direct parasitological testing and/or clinical suspicion. Starting in 2009, the introduction of serological tests resulted in an increase in the number of diagnosed cases. Moreover, after 2015 patients with risk criteria were screened for infection. The combination of serology testing and the record of some simple data such as country of origin, haematuria in childhood or eosonophil count, tripled the number of diagnoses (see “Fig 2”).

Our results compel us to question the true degree of underdiagnosis in our country. Quite possibly the disease is far more common than presumed. However, probably because of its insidious clinical presentation, the low degree of knowledge about the disease and the absence of accessible tests, family practitioners have few tools to diagnose schistosomiasis.

Chronic and irreversible complications, especially renal failure, were present in a high percentage of patients included in this study. We emphasize that in the 6 patients with terminal renal failure the diagnosis of schistosomiasis was made when the structural damage was already irreversible. In this phase of infection we often could not discern the species of *Schistosoma* involved since renal failure is a complication that can be due not only of *S*. *haematobium* but also *S*.*mansoni*, secondary to glomerular damage [18]. The importance of starting treatment as early as possible must be highlighted, particularly in those patients awaiting renal transplantation, given the possibility of recrudescence of the disease in the transplanted kidney [19, 20]. It would be reasonable to implement *Schistosoma* serology screening for all those patients coming from endemic countries and suffering renal insufficiency of unknown origin.

The low number of *Schistosoma* infections identified among women (only 5 which represent a 8.2%) is striking considering that prevalences in endemic countries do not show significant differences by gender [16, 21, 22]. The reason is not known but it may be related to the higher proportion of male sub-Saharan immigrants compared with women. Other possible factors might be that the presence of urinary symptoms are more common and more nonspecific in women than in men, or the confounding role of gynecological symptoms [23,24].

Co-infection with HBV (19.7% of patients) is a demonstrable risk factor for developing liver disease earlier and to more serious degree [25]. Therefore, screening for the hepatitis B virus (and also hepatitis C virus) should be integrated into the management of these patients.

Regarding the economic aspects of the disease, 9 out of 22 patients diagnosed with chronic organ complications generated a direct health expenditure of €369,230/year; 6 of them underwent hemodialysis (€23,620/person/year) with kidney transplantations in 3 (€17,158/transplant), not to mention the costs associated with medication and hospital admissions [26]. In contrast, the pharmacological treatment of the 22 patients treated with praziquantel amounted to €2,200. An earlier diagnosis would surely have avoided the high cost of treating complications. The difficulty of diagnosis in the chronic phase of the infection points to the urgency of upgrading diagnostic methods by means of specialized devices. Serology, the standard screening method, has a sensitivity of over 90% for travelers and slightly less for people originally from endemic countries [27]. Antibody-based assays are quite sensitive but cannot distinguish history of exposure from active infections, and the results can also reflect cross-reaction with other platyhelminths. Therefore, among individuals native to *Schistosoma*-endemic areas the implementation of antigenic or molecular techniques should be considered [28]. Recent studies advocate the use of these diagnostic methods in patients with presumed exposure such as long-standing immigrants [29–33]. Such techniques would make it possible to differentiate between active disease from chronic or treated schistosomiasis. Thus far, the outcome of treatment has been based on indirect markers such as normalization of eosinophilia and liver enzymes which are highly unreliable.

The present study assesses African immigrant community long established in the EU. The number of similar articles is very limited, and its singularity could be considered its main strength. When analyzing sub-categories, however, the number of patients is actually quite small; more and larger samples are needed in order to be able to draw categorical conclusions.

The Mediterranean area is a former habitat of *Bulinus* snails and, furthermore, climate warming could spread the conditions that favor local genitourinary schistosomiasis transmission to southern Europe. In 2013, an unexpected outbreak of urogenital schistosomiasis occurred in Corsica, France, with more than 120 local people or tourists infected. Molecular sequence data analysis showed that the Corsican schistosomes were closely related to those from Senegal, in West Africa. The emergence of urinary schistosomiasis in Corsica suggests that schistosomiasis could be a cause for concern in a Mediterranean *Bulinus*-colonized Europe [34]. Whether the source of the outbreak in Corsica was sub-clinical infected immigrants or not, remains unclear; nevertheless, a more comprehensive EU-wide approach, including screening of high-probability communities, seems advisable.

Several factors play a role in the reality of schistosomiasis in non-endemic countries producing a delay in the diagnose, specially the high percentage of complications,the low clinical suspicion rate and the difficulty of diagnosis. The present study highlights the need to improve management and earlier detection of the disease through the implementation of screening protocols among populations at risk.

In short, long-term schistosomiasis remains a problem even in an area previously identified as of high prevalence among immigrants. We therefore recommend the introduction of a screening program based on first a questionnaire about symptoms and signs of active infection and then serological testing.
